# Supplementary material for: Degenerative Joint Damage Is Not a Risk Factor for Streptococcal Sepsis and Septic Arthritis in Mice
Source: Life (Basel). 2021 Aug 5;11(8):794. doi: 10.3390/life11080794 (PMC8400161; doi:10.3390/life11080794)
Supplement: Supplementary file 1 [file life-11-00794-s001.zip › life-1272950-supplementary.pdf]

life

MDPI

Communication

Supplementary Information: Degenerative Joint Damage Is Not a Risk Factor for Streptococcal Sepsis and Septic Arthritis in Mice

Johann Volzke * and Brigitte Müller-Hilke

Core Facility for Cell Sorting and Cell Analysis, University Medical Center Rostock, 18057 Rostock, Germany; zsa@med.uni-rostock.de
* Correspondence: johann.volzke@med.uni-rostock.de; Tel.: +49-381-494-5881

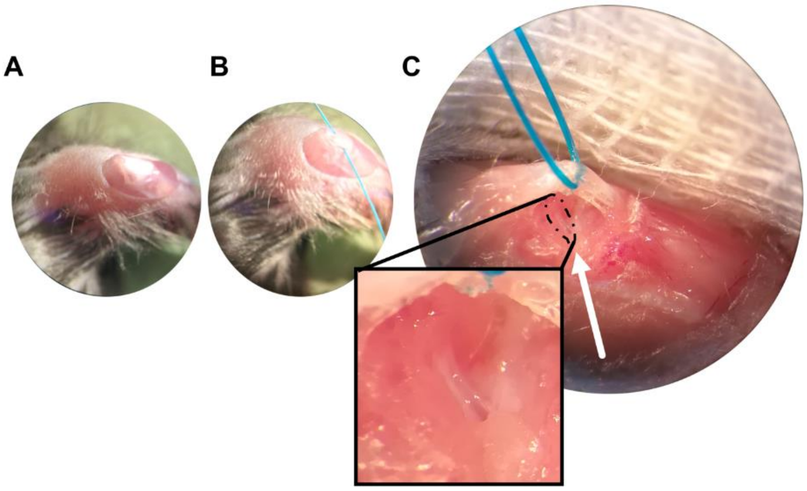

Figure S1. Procedure of the anterior cruciate ligament transection (ACLT) under a stereomicroscope. (A) The patella is exposed after removal of the skin. (B) Subsequent to a medial arthrotomy, a synthetic suture is guided under the patella. (C) The joint capsule is laid open after stretching the patella with the suture. Subsequently, the infrapatellar fat pad is removed and the anterior cruciate ligament becomes visible for transection.

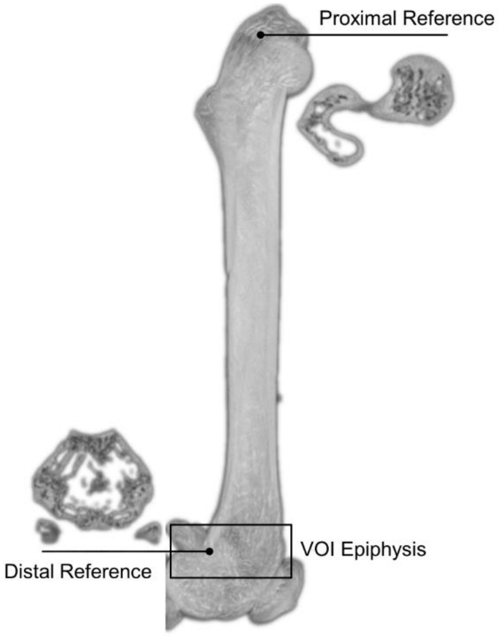

Figure S2. Selection of femoral reference points and volume of interest for the bone morphometric analyses via micro-computed tomography. The proximal reference level was selected at the fusion of greater trochanter and femoral head. The distal reference level was located at the metaphyseal growth plate where low density cartilage merges with bone primary spongiosa. The length of the bone was normalized to the reference levels and µCT analyses of cancellous and cortical bone were performed at ± 5% of the femoral epiphysis and at 10% of mid diaphysis, respectively.

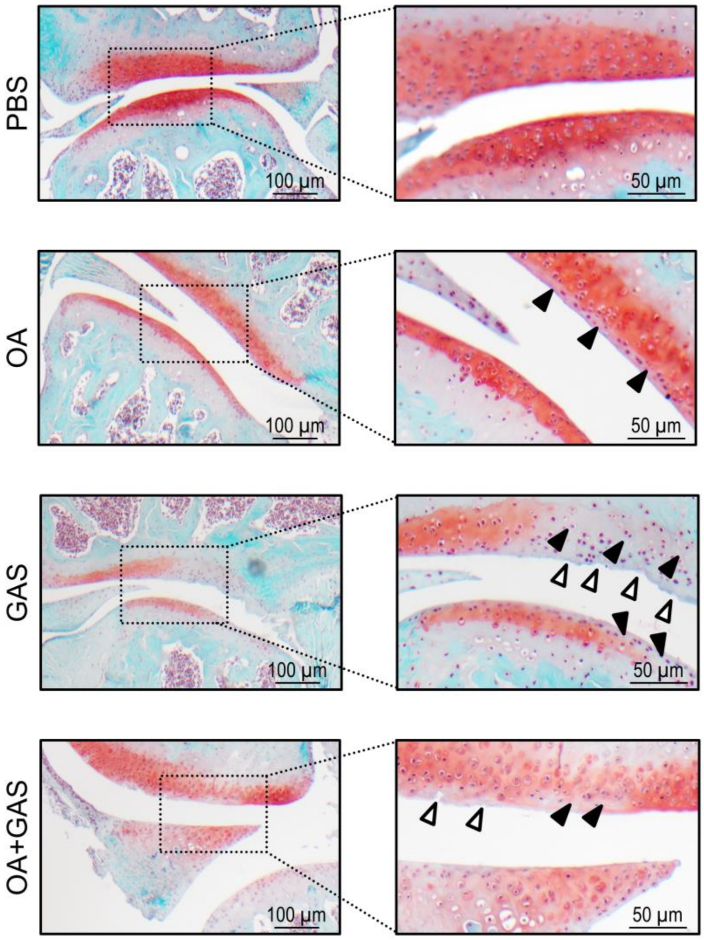

Figure S3. Osteoarthritis and GAS infection induced the erosion of cartilage and the degradation of extracellular matrix. Images of histological sections are shown. The staining of proteoglycans was performed with Safranin O (red color). Fast Green (green color) and hematoxylin (blue color) were used as counterstains. The structure of the tibiofemoral cartilage from healthy C57BL/6J mice (PBS, representative for n = 4) was intact as demonstrated by consistent proteoglycan staining and a smooth surface. The cartilage of mice 15 weeks after the anterior cruciate ligament transection (OA, representative for n = 5) exhibited a slight loss of extracellular matrix (black arrowheads). In a severe case of knee joint infection (GAS), the surface of the cartilage was found to be eroded (white arrowheads) which was associated with a considerable loss of proteoglycans. The cartilage of the knee joint from an infected animal with the preceding degenerative joint disease (OA+GAS) also manifested a deteriorated surface including the loss of Safranin O staining.

Table S1. Bone morphometry at the femoral epiphysis of GAS infected or non-infected STR/ort mice.

		**PBS (n = 14)** **Median (IQR)**	**GAS (n = 22)** **Median (IQR)**	*p*
	Tt.Ar [mm^2]	3.79 (3.33–4.01)	3.64 (3.35–4.50)	0.67
	Ct.Ar [mm^2]	3.46 (3.02–3.66)	3.35 (3.06–4.14)	0.69
	Ct.Ar/Tt.Ar [%]	91.1 (90.5–91.8)	91.3 (90.8–92.0)	0.62
Cortical Bone	Ct.Th [mm]	0.256 (0.242–0.281)	0.277 (0.248–0.305)	0.28
	J [mm^4]	4.00 (3.62–4.40)	4.06 (3.55–4.55)	0.84
	Ct.Pm [mm]	25.3 (24.4–26.7)	25.7 (23.9–26.6)	0.89
	ε	0.549 (0.461–0.613)	0.488 (0.447–0.523)	0.089

	Φ [%]	2.56 (1.47–4.44)	2.02 (1.12–2.46)	0.14
Trabecular Bone	TV [mm³]	3.02 (2.71–3.71)	3.04 (2.74–3.33)	0.49
	BV [mm³]	1.46 (1.37–1.68)	1.55 (1.51–1.66)	0.57
	BV/TV [%]	47.2 (45.5–48.6)	51.5 (49.7–56.9)	0.005
	BS [mm²]	54.1 (51.7–59.1)	57.4 (55.3–58.4)	0.57
	BS/BV [mm⁻¹]	35.6 (34.9–37.0)	36.2 (34.2–38.0)	0.74
	BS/TV [mm⁻¹]	16.7 (16.5–17.8)	18.3 (17.2–21.4)	0.007
	SMI	1.01 (0.848–1.12)	0.913 (0.697–0.992)	0.24
	Tb.Pf [mm⁻¹]	5.89 (4.96–6.60)	5.36 (4.11–6.63)	0.47
	Tb.Th [mm]	0.108 (0.105–0.113)	0.106 (0.105–0.110)	0.51
	Tb.N [mm⁻¹]	4.37 (4.23–4.51)	4.88 (4.52–5.31)	0.0056
	Tb.Sp [mm]	0.193 (0.167–0.205)	0.175 (0.160–0.193)	0.21
	Γ	652 (599–677)	670 (639–739)	0.16

Tt.Ar—Total cross-sectional area inside the periosteal envelope. Ct.Ar—Cortical bone area. Ct.Ar/Tt.Ar—Cortical area fraction. Ct.Th—Cortical thickness. J—Polar moment of inertia. Ct.Pm—Cortical perimeter. ε—Eccentricity. Φ—Closed Porosity. TV—Total Volume. BV—Bone Volume. BV/TV—Bone volume fraction. BS—Bone Surface. BS/BV—Specific Bone Surface. BS/TV—Bone Surface Density. SMI—Structure model index. Tb.Pf—Trabecular pattern factor. Tb.Th—Trabecular thickness. Tb.N—Trabecular number. Tb.Sp –Trabecular Spacing. Γ—Connectivity.

Table S2. Bone morphometry at the femoral epiphysis of C57BL/6J mice 14 weeks after ACLT or sham surgery (naïve).

		Naïve (n = 11) Median (IQR)	ACLT (n = 5) Median (IQR)	*p*
Cortical Bone	Tt.Ar [mm²]	4.25 (3.73–4.45)	3.40 (3.18–3.66)	0.22
	Ct.Ar [mm²]	3.92 (3.48–4.14)	3.08 (2.91–3.38)	0.15
	Ct.Ar/Tt.Ar [%]	92.2 (91.2–92.9)	91.0 (90.7–91.3)	0.22
	Ct.Th [mm]	0.288 (0.271–0.293)	0.243 (0.241–0.244)	0.038
	J [mm⁴]	4.75 (4.01–5.97)	3.90 (3.71–4.12)	0.18
	Ct.Pm [mm]	27.0 (24.1–28.5)	25.2 (24.1–25.6)	0.74
	ε	0.611 (0.518–0.649)	0.434 (0.431–0.482)	0.0032
	Φ [%]	5.02 (3.85–6.54)	6.20 (5.57–6.32)	0.74
Trabecular Bone	TV [mm³]	3.35 (2.91–3.65)	3.83 (3.39–3.87)	0.069
	BV [mm³]	1.86 (1.67–2.01)	2.06 (1.84–2.07)	0.38
	BV/TV [%]	55.6 (54.6–57.6)	53.7 (53.5–54.3)	0.038
	BS [mm²]	66.6 (61.3–71.7)	69.7 (64.6–72.8)	0.58
	BS/BV [mm⁻¹]	36.1 (34.7–37.0)	34.4 (33.9–35.1)	0.052
	BS/TV [mm⁻¹]	20.0 (19.8–20.7)	18.8 (18.2–18.9)	0.00046
	SMI	0.776 (0.481–0.900)	0.489 (0.405–0.579)	0.15
	Tb.Pf [mm⁻¹]	4.733 (2.81–5.50)	2.73 (2.35–3.39)	0.15
	Tb.Th [mm]	0.106 (0.104–0.109)	0.107 (0.106–0.107)	0.74
	Tb.N [mm⁻¹]	5.38 (5.11–5.58)	5.06 (4.73–5.07)	0.028
	Tb.Sp [mm]	0.140 (0.132–0.149)	0.153 (0.151–0.155)	0.11
	Γ	767 (661–850)	873 (731–888)	0.51

Table S3. Septic arthritis and sepsis disease activities, as well as bacterial burden in blood, liver, spleen and knee joint capsules of infected C57BL/6J mice without (GAS) and with osteoarthritis (OA+GAS), respectively. CFU: colony-forming unit.

	GAS Median (IQR)	OA+GAS Median (IQR)	*p*
Arthritis Score	0 (0–4)	0 (0–0)	0.39
Sepsis Score	18 (6–25)	8 (0–14)	0.12
CFU blood [mL⁻¹]	2.0×10^2 (1.0×10^2–1.4×10^5)	1.0×10^2 (1.0×10^2–6.3×10^3)	0.47

CFU liver [mL^{-1}]	5.4×10^3 (1.0×10^2–7.5×10^6)	1.0×10^2 (1.0×10^2–1.4×10^5)	0.32
CFU spleen [mL^{-1}]	2.7×10^3 (1.0×10^2–6.3×10^6)	1.0×10^2 (1.0×10^2–1.1×10^5)	0.17
CFU knee [mL^{-1}]	$< 5 \times 10^2$ (5×10^2–5×10^2)	$< 5 \times 10^2$ (5×10^2–5×10^2)	0.52

Table S4. Bone morphometry at the femoral epiphysis after GAS infection of C57BL/6 mice with and without ACLT.

		**GAS (n = 30)** **Median (IQR)**	**OA+GAS (n = 7)** **Median (IQR)**	***p***
Cortical Bone	Tt.Ar [mm^2]	4.50 (3.82–4.70)	5.22 (4.68–5.33)	0.11
	Ct.Ar [mm^2]	4.13 (3.50–4.42)	4.75 (4.13–4.92)	0.17
	Ct.Ar/Tt.Ar [%]	92.3 (91.7–93.0)	91.1 (90.6–92.5)	0.26
	Ct.Th [mm]	0.312 (0.265–0.331)	0.311 (0.292–0.324)	0.98
	J [mm^4]	5.29 (4.51–6.57)	6.20 (5.59–7.83)	0.23
	Ct.Pm [mm]	27.1 (25.3–30.1)	30.6 (27.3–34.1)	0.13
	ε	0.607 (0.536–0.650)	0.526 (0.413–0.635)	0.35
	Φ [%]	3.82 (2.24–5.55)	3.82 (1.99–3.87)	0.53
Trabecular Bone	TV [mm^3]	3.23 (2.71–3.82)	3.61 (3.15–4.38)	0.37
	BV [mm^3]	1.92 (1.67–2.11)	2.03 (1.86–2.45)	0.39
	BV/TV [%]	57.6 (55.0–60.1)	56.2 (55.9–58.0)	0.69
	BS [mm^2]	67.8 (60.4–74.7)	75.2 (66.0–81.3)	0.39
	BS/BV [mm^{-1}]	36.2 (34.6–37.1)	33.5 (33.2–37.5)	0.33
	BS/TV [mm^{-1}]	20.8 (19.1–22.2)	18.8 (18.6–21.9)	0.48
	SMI	0.683 (0.536–0.820)	0.812 (0.700–0.872)	0.33
	Tb.Pf [mm^{-1}]	4.07 (3.04–5.38)	4.53 (4.06–5.21)	0.56
	Tb.Th [mm]	0.106 (0.104–0.109)	0.117 (0.104–0.118)	0.16
	Tb.N [mm^{-1}]	5.48 (5.07–5.69)	4.79 (4.72–5.56)	0.13
	Tb.Sp [mm]	0.144 (0.135–0.150)	0.192 (0.137–0.211)	0.17
	Γ	727 (692–848)	730 (655–846)	0.86
